# Emergency Department Presentations following Tropical Cyclone Yasi

**DOI:** 10.1371/journal.pone.0131196

**Published:** 2015-06-25

**Authors:** Peter Aitken, Richard Charles Franklin, Jenine Lawlor, Rob Mitchell, Kerrianne Watt, Jeremy Furyk, Niall Small, Leone Lovegrove, Peter Leggat

**Affiliations:** 1 College of Public Health, Medical and Veterinary Sciences, James Cook University, Queensville, Townsville, Australia; 2 Emergency Department, The Townsville Hospital, Townsville, Queensville, Australia; 3 Royal Life Saving Society - Australia, Sydney, Australia; Curtin University, AUSTRALIA

## Abstract

**Introduction:**

Emergency departments see an increase in cases during cyclones. The aim of this study is to describe patient presentations to the Emergency Department (ED) of a tertiary level hospital (Townsville) following a tropical cyclone (Yasi). Specific areas of focus include changes in: patient demographics (age and gender), triage categories, and classification of diseases.

**Methods:**

Data were extracted from the Townsville Hospitals ED information system (EDIS) for three periods in 2009, 2010 and 2011 to coincide with formation of Cyclone Yasi (31 January 2011) to six days after Yasi crossed the coast line (8 February 2012). The analysis explored the changes in ICD10-AM 4-character classification and presented at the Chapter level.

**Results:**

There was a marked increase in the number of patients attending the ED during Yasi, particularly those aged over 65 years with a maximum daily attendance of 372 patients on 4 Feb 2011. The most marked increases were in: Triage categories - 4 and 5; and ICD categories - diseases of the skin and subcutaneous tissue (L00-L99), and factors influencing health care status (Z00-Z99). The most common diagnostic presentation across all years was injury (S00-T98).

**Discussion:**

There was an increase in presentations to the ED of TTH, which peaked in the first 24 – 48 hours following the cyclone and returned to normal over a five-day period. The changes in presentations were mostly an amplification of normal attendance patterns with some altered areas of activity. Injury patterns are similar to overseas experience.

## Introduction

In Australia over the last 150 years, cyclones (hurricane) are estimated to have caused the deaths of approximately 2000 people, with a financial cost in excess of 6 trillion dollars.[1] Approximately 119 million people are affected by an average of 80–90 individual cyclone systems each year,[2, 3] making them one of the most common natural disasters across all continents.[4] Historically, developing nations of the Asia-Pacific region have experienced the greatest absolute and proportionate mortality from tropical cyclones. [2]

The severity of cyclones is rated using a five category system based on wind velocity,[5] with category four and five cyclones expected to achieve wind gusts in excess of 225 km/h and 280km/h respectively resulting in significant structural damage. Cyclone Tracy (Category (Cat) 5, Darwin, 1974), resulted in 71 deaths and approximately 650 injuries.[6] More recently Cyclone Larry (Cat 5, Queensland (Qld), 2006), Cyclone George (Cat 5, Western Australia (WA), 2008) and Cyclone Yasi (Cat 5, Qld, 2011) have all resulted in widespread damage. In the United States (US) two recent memorable hurricanes (Katrina and Sandy) have caused approximately US$174 billion in damage [7, 8].

During cyclones hospitals often see a dramatic increase in the number of patients attending the emergency department [9]. As a consequence, considerable attention has been given to surge capability. A full understanding of the epidemiology of cyclones including patterns of injury and illness, can inform emergency preparedness and planning. Studies are required to describe the epidemiology of cyclone related injuries in terms of timing and type of injury and degree of impact on patients with co-morbidities.

The aim of this study is to describe the patient presentations to the Emergency Department (ED) of a tertiary level hospital (Townsville) following a tropical cyclone (Yasi). Specific areas of focus include changes in: patient demographics (age and gender), triage categories, and classification of diseases.

### Setting

The Townsville Hospital (TTH) is the tertiary referral centre for North Queensland, Australia, which has a population of approximately 700,000 people and an area of 750,000 square km. It also serves an immediate population of approximately 227,000 and is the secondary referral centre for a number of local facilities—the closest of which are all approximately 100 km away [10]. TTH has all services, including neurosurgery, cardiothoracic surgery, haematological and radiation oncology as well as a mixed adult and paediatric ICU and neonatal ICU. The exceptions are burns and spinal units, which are transferred 1300 km south to Brisbane, which is also the location of the nearest alternative tertiary level facility. The Emergency Department annual census is approximately 68,000 with a 30% admission rate. TTH ED is the only ED in Townsville.

### Cyclone Yasi

**Fig 1:**
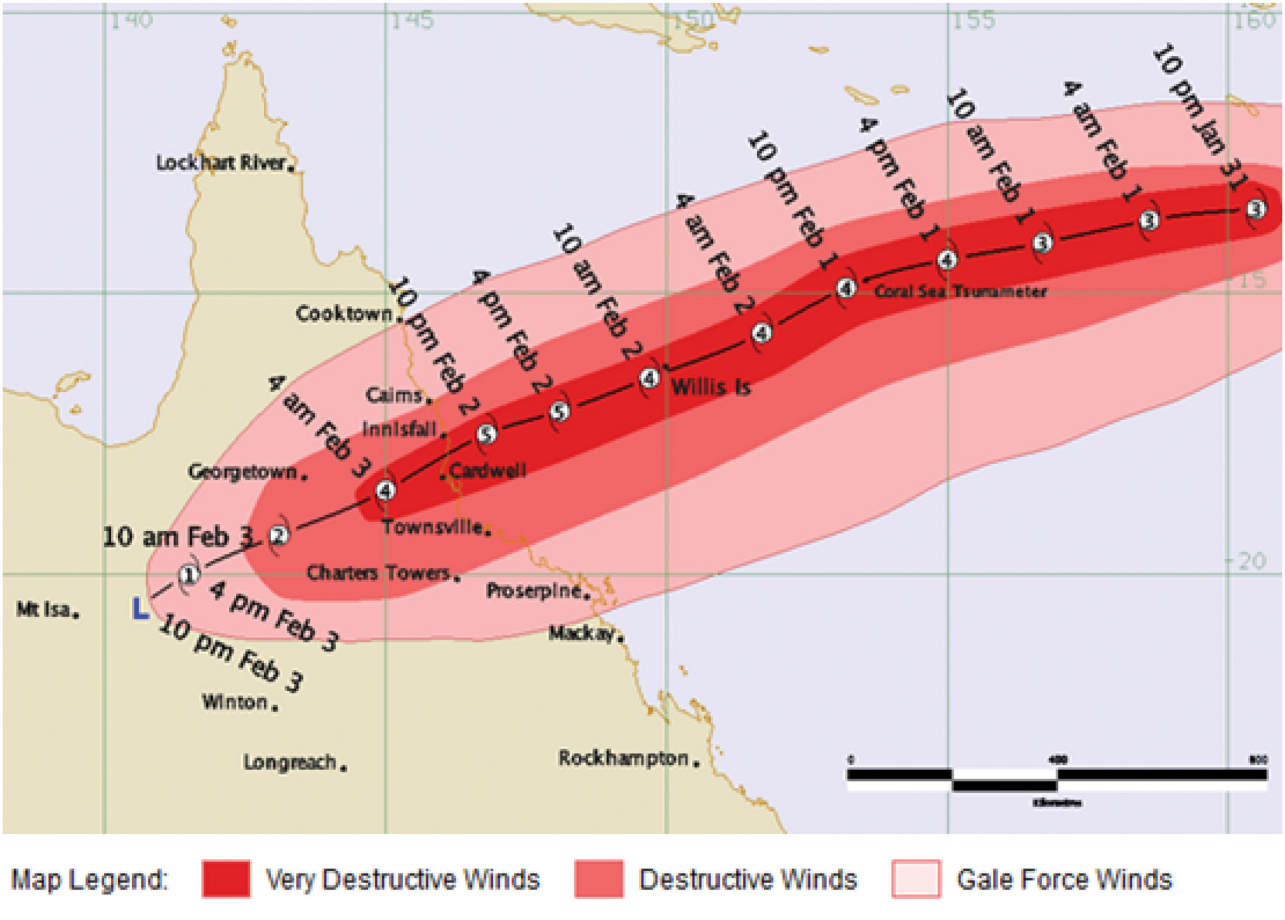
Tropical Cyclone Yasi track and intensity information, 10pm 31 January 2011 to 10pm 3 February 2011. Source: Australian Government, Bureau of Meteorology http://www.bom.gov.au/cyclone/history/yasi.shtml#track (accessed 17 January 2013) [11].

Tropical Cyclone (TC) Yasi was the largest cyclone to cross the Australian coastline in recorded history with a destructive core more than 100 km wide and Category 5 wind speeds [11]. Yasi followed TC Anthony, was generated in Fijian waters and travelled over a three day period toward the coast while being tracked by the Bureau of Meteorology (http://www.bom.gov.au/cyclone/history/yasi.shtml#track) [11]. A little after Midnight on Thursday 3 February, Yasi crossed the Queensland coastline near Mission Beach. As can be seen in the figure Townsville experienced conditions similar to a category 3 cyclone and significant levels of rain. [11] (Fig 1).

Yasi caused considerable disruption to local infrastructure as a result of wind damage and flooding. Part of the population were evacuated, particularly those in low lying areas and 85% of the city lost power for at least 24 hours with approximately 60,000 homes waiting over a week for power to be restored. As a new structure built to cyclone standards, TTH was able to act as a shelter and remain functioning throughout, utilising generator power. The Hospital Mass Casualty plan was activated which included expansion of the ED into the adjacent Women’s and Children clinic area to provide additional space for care of lower acuity patients, all of which are included in the ED attendance data. The timeline of events is shown in Table 1.

**Table 1:** Timeline of Cyclone Yasi.

Date	Time	Event
28^th^ Jan 2011	1149	Bureau of Meterology (BoM) issues warning that Cyclone Anthony reforming
30^th^ Jan 2011		Tropical Cyclone Anthony crosses as Cat 2
31^st^ Jan 2011	1213	Alert for Yasi—preparation and warnings for coastal and low lying areas
1^st^ Feb 2011		Police commence door knocking
2^nd^ Feb 2011	1015	Landfill and transfer stations close
2^nd^ Feb 2011	1400	Road closures for general motorists
2^nd^ Feb 2011	2000	Road closures for emergency medical services (EMS)
2^nd^ Feb 2011	2230	89,000 homes without power
2^nd^ Feb 2011	Midnight	Yasi Makes Landfall just after midnight
3^rd^ Feb 2011	0600	TTH Mass Casualty Plan activated
3^rd^ Feb 2011		Only 15% of homes have power
3^rd^ Feb 2011	0930	Assessment of road condition starts, roads reopened to residents throughout the day
9^th^ Feb 2011		Power restored to all homes in Townsville (still some power out in the region)
6^th^ Feb 2011		Schools re-open

## Methods

Data were extracted from The Townsville Hospital Emergency Department Information Systems (EDIS) for the three periods, 1 January 2009 to 31 March 2009, 1 January 2010 to 31 March 2010, 1 January 2011 to 31 March 2011. The three month period was used to show trend over time.

**Fig 2:**
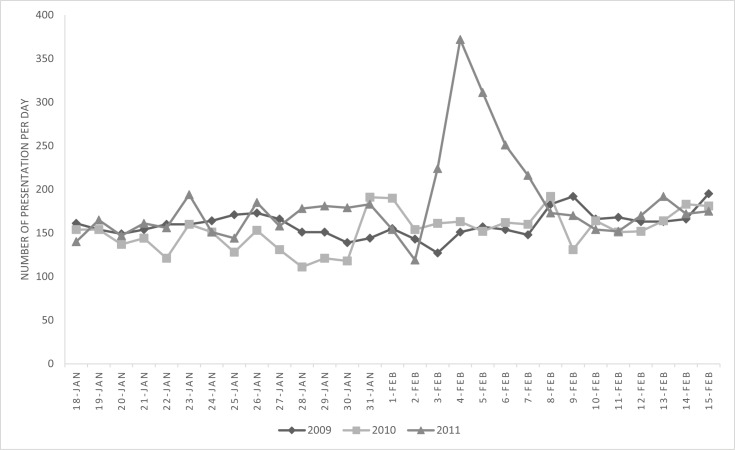
Townsville Emergency Department Presentations number by day, 18 January to 15 February, 2009–2011.

The study period was from 0000 hrs on 31-January-2011 when Yasi was forming to the 8^th^ February 2011. This period includes 2 days before the cyclone crossed the coast (2^nd^ February 2011) to 6 days after (8^th^ February 2011) by which stage patient presentations had returned to normal numbers (Fig 2). EDIS was used to collect data on overall patient presentation numbers as well as the arrival date and time, gender, age, triage category (Australasian Triage Scale), diagnosis (coded to ICD10), departure destination, date and time.

The years 2010 and 2009 were used as comparison years. To allow for existing variation between weekday and weekend activity the 2011 year was matched to the previous year’s same day of the week. This meant that there was a difference of 1 calendar day in the date for each year.

### Diagnostic codes used to identify

Diagnosis was coded to ICD10 ICD 4-character classification, and presented at the Chapter level for ICD.

### Statistical analysis

Analysis was undertaken using IBM SPSS Statistics Version 19, Pearson’s Chi-Square test was undertaken to determine statistical significance for categorical variables. When examining the different demographic descriptors and ICD-10 codes we looked for a greater than 50% average daily variation from the baseline level of increased activity against both comparison years. Average daily presentations were based on the 9 day (ie 31 Jan to 8 Feb) study period. A 50% increase was arbitrarily chosen as it was greater than the 40% average change seen in this study and the authors believed that this would be beyond the normal day-to-day variation (except for categories with small numbers, these were reviewed separately).

### Ethics

Ethics approval was obtained from the Townsville Health Service District Human Research Ethics Committee (HREC) (Number HREC/11/QTHS/77) and James Cook University HREC (number H4230). The data were extracted from the administrative database EDIS by the hospital, the data were anonymized and de-identified prior to being provided to the researchers for analysis. Consent for the patients was not required for this study as the data were de-identified and retrospective and there was no disadvantage to the participant or their relatives or to any collectively involved; also due to the large numbers of patients, it would also be impossible in practice to obtain consent and would prejudice the scientific value of the research[12].

## Results

There was a marked increase in numbers of patients attending the TTH ED during the study period. The increase in presentations occurred over a 5 day period (3^rd^- 7^th^ February) and peaked (372 patients) on 4^th^ February. This resulted in mean daily presentations for the study period increasing from 158 (2009) and 162 (2010) to 222 (Yasi) per day. (Fig 2)

**Fig 3:**
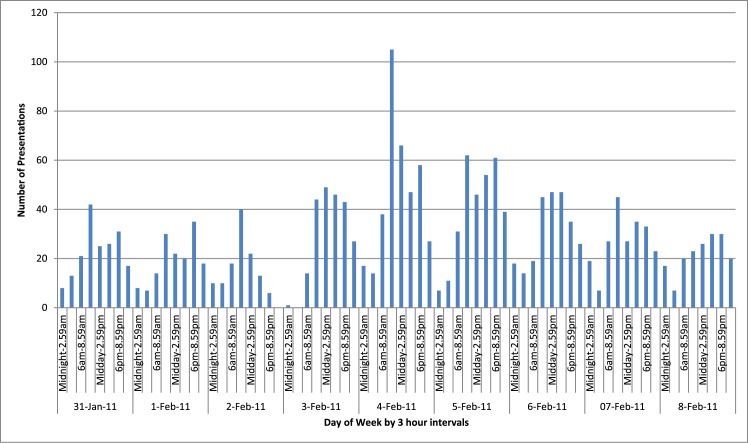
Townsville Emergency Department presentations by 3 hour periods and day, 31-Jan-2011 to 8-Feb-2011.

The temporal pattern of attendance in three-hour blocks (Fig 3) showed the attendance pattern was mostly maintained with peaks still occurring in the morning (0900–1200) and evening (1800–2100) and decreased attendances overnight (2400–0600). Only one patient presented between 2020 hours on the 2^nd^ February and 0700 hours on 3^rd^ February, which correlates with the period of road closure for EMS (Table 1 and Fig 3). The peak attendance period was 0900–1200 on 4^th^ February, 36 hours after Yasi passed through. (Fig 3)

Overall there was an approximately 40% increase in daily activity (36.7% vs 2010; 41.0% vs 2009) (Table 2). When examining the different demographic descriptors and ICD-10 codes we looked for a greater than 50% average daily variation from the baseline level of increased activity (i.e. 36.7% in 2010 and 41% in 2009). Thus increases in presentations of at least 55% compared to 2010 and more than 61.5% compared to 2009 were regarded as important. Similarly decreases, or an increase less than 18.4% compared to 2010 and 20.5% compared to 2009 were regarded as important. These changes had to be present in both comparison years.

**Table 2:** Average presentation per day during Yasi and years prior by gender, age, indigenous status, triage category and discharge status, 31-Jan-2011 to 8-Feb-2011, TTH ED.

	*Yasi*	*2010 (1 Year Prior to Yasi)*	*2009 (2 Years Prior to Yasi)*	*Percentage Change between Yasi and 2010*	*Percentage Change between Yasi and 2009*
Av per Day	222.6	162.8	157.9	36.7	41.0
Gender					
Male	114.1 (*51*.*3%)*	84.4 (*51*.*9%)*	81.7 (*51*.*8%)*	35.1	39.7
Female	108.4 (*48*.*7%)*	78.3 (*48*.*1%)*	76.2 (*48*.*3%)*	38.4	42.3
Age Group					
0–15 years	45.8 (*20*.*6%)*	32.6 (*20*.*0%)*	34.9 (*22*.*2%)*	40.6	31.2
16–24 Years	31.7 (*14*.*2%)*	27.4 (*16*.*8%)*	26.9 (*17*.*0%)*	15.4	17.8
25–34 Years	28.0 (*12*.*6%)*	24.6 (*15*.*1%)*	22.4 (*14*.*2%)*	14.0	24.8
35–44 Years	31.6 (*14*.*2%)*	23.1 (*14*.*2%)*	23.4 (*14*.*8%)*	36.5	34.6
45–54 Years	25.8 (*11*.*6%)*	18.7 (*11*.*5%)*	17.8 (*11*.*3%)*	38.1	45.0
55–64 Years	23.0 (*10*.*3%)*	14.4 (*8*.*9%)*	13.6 (*8*.*6%)*	59.2	69.7
65+ years	36.8 (*16*.*5%)*	22.0 (*13*.*5%)*	18.9 (*12*.*0%)*	67.2	94.7
Indigenous Status					
Aboriginal and or Torres Strait Islander	26.0 (11.7%)	22.4 (13.8)	23.7 (15%)	15.8	9.9
Not Indigenous	195.2 (*87*.*7%)*	140.1 (*86*.*1%)*	133.4 (*84*.*5%)*	39.3	46.3
Not Stated / Unknown	1.3 (*0*.*6%)*	0.2 (*0*.*1%)*	0.8 (*0*.*5%)*	500.0	71.4
Triage Category					
1	0.9 (*0*.*4%)*	1.4 (*0*.*9%)*	1.3 (*0*.*8%)*	-38.5	-33.3
2	17.9 (*8*.*0%)*	16.2 (*10*.*0%)*	14.0 (*8*.*9%)*	10.3	27.8
3	69.9 (*31*.*4%)*	61.4 (*37*.*7%)*	51.2 (*32*.*4%)*	13.7	36.4
4	117.4 (*52*.*7%)*	78.7 (48.3%)	80.0 (*50*.*1%)*	49.3	46.8
5	16.4 (*7*.*4%)*	5.0 (*3*.*1%)*	11.3 (*7*.*2%)*	228.9	45.1
Discharge status					
Admitted (excl. ED bed)	58.0 (*26*.*1%)*	47.6 (*29*.*5%)*	33.9 (*21*.*5%)*	22.0	71.1
Did not wait	10.7 (*4*.*8%)*	8.3 (*5*.*1%)*	5.6 (*3*.*6%)*	28.0	92.0
Died in ED	0.1 (*0*.*1%)*	0.0 (*0*.*0%)*	0.0 (*0*.*0%)*		
ED service event completed—discharged	146.0 (*65*.*6%)*	98.6 (*60*.*6%)*	114.2 (*72*.*3%)*	48.1	27.8
Left after treatment commenced	7.1 (3.2%)	6.9 (*4*.*2%)*	2.7 (*1*.*7%)*	3.2	166.7
Transfer to another hospital	0.7 (*0*.*3%)*	1.4 (*0*.*9%)*	1.6 (*0*.*1%)*	-53.8	-57.1

There were increased attendances by both males and females, and although there was a slightly larger, but non-significant (Χ^2^ = .14 p = .933), increase in females presenting the male to female attendance ratio was maintained (2011 = 1.05:1.00; 2010 = 1.08:1.00; 2009 = 1.07:1.00).

There were increased attendances across all age groups, but this was proportionally more for the elderly, with increasing attendances noted with advancing age (Χ^2^ = .14 p = .003). While the largest group of patients attending was those aged 0–15 years this was consistent across all years and also consistent with the general levels of increased activity. Those aged 16–24 and 25–34 years showed the least increase in attendances and well below that of the daily average, in both comparison years for those aged 16–24 years. The largest proportional increase in attendance was for those aged more than 65 years with levels of importance (based on greater than 50% variation) also reached for the 55–64 year age group. (Table 3)

**Table 3:** Average presentation per day during Yasi and years prior by ICD-10-AM group classifications, 31-Jan-2011 to 8-Feb-2011, TTH ED.

	*Yasi*	*2010 (1 Year Prior to Yasi)*	*2009 (2 Years Prior to Yasi)*	*Percentage Change between Yasi and 2010*	*Percentage Change between Yasi and 2009*
*Average per day*	*222*.*6*	*162*.*8*	*157*.*9*	*36*.*7*	*41*.*0*
Certain Infectious and Parasitic Diseases (A00-B99)	13.2 (*5*.*9%)*	5.7 (*3*.*5%)*	10.6 (*6*.*7%)*	133.3	25.3
Neoplasms (C00-D48) and Diseases of Blood and Blood-Forming Organs (D50-D89)	2.6 (1.2*%)*	1.3 (*0*.*9%)*	1.7 (1.0%)	91.7	53.3
Endocrine, nutritional and metabolic diseases (E00-E89)	2.3 (*1*.*0%)*	3.4 (*2*.*1%)*	1.7 (*1*.*1%)*	-32.3	40.0
Mental and Behavioural Disorders (F00-F99)	5.6 (*2*.*5%)*	7.0 (*4*.*3%)*	7.4 (*4*.*7%)*	-20.6	-25.4
Diseases of the nervous system (G00-G99)	3.3 (*1*.*5%)*	2.7 (*1*.*7%)*	2.4 (*1*.*6%)*	25.0	36.4
Diseases of the eye and adnexa (H00-H59)	3.1 (*1*.*4%)*	1.2 (*0*.*7%)*	1.0 (*0*.*6%)*	154.5	211.1
Diseases of the ear and mastoid process (H60-H95)	4.0 (*1*.*8%)*	3.1 (*1*.*9%)*	3.3 (*2*.*1%)*	28.6	20.0
Diseases of the circulatory system (I00-I99)	6.0 (*2*.*7%)*	5.1 (*3*.*1%)*	4.2 (*2*.*6%)*	17.4	42.1
Diseases of the respiratory system (J00-J99)	16.0 (*7*.*2%)*	8.9 (*5*.*5%)*	13.6 (*8*.*6%)*	80.0	18.0
Diseases of the digestive system (K00-K93)	11.7 (*5*.*3%)*	10.8 (*6*.*6%)*	8.8 (*5*.*6%)*	8.2	32.9
Disease of the skin and subcutaneous tissue (L00-L99)	13.3 (*6*.*0%)*	5.1 (*3*.*1%)*	5.3 (*3*.*4%)*	160.9	150.0
Diseases of the musculoskeletal system and connective tissue (M00-M99)	5.2 (*2*.*3%)*	3.6 (*2*.*2%)*	3.4 (2.2%)	46.9	51.6
Diseases of the Genitourinary system (N00-N99)	10.3 (*4*.*6%)*	9.2 (*5*.*7%)*	9.4 (*6*.*0%)*	12.0	9.4
Pregnancy, child birth and the puerperium (O00-O99)	2.6 (*1*.*2%)*	2.2 (*1*.*4%)*	2.7 (*1*.*8%)*	15.0	-4.2
Symptoms, signs and abnormal clinical and laboratory findings, NEC (R00-R99)	19.8 (*8*.*9%)*	22.9 (*14*.*1%)*	21.3 (13.5%)	-13.6	-7.3
Injury, poisoning and certain other consequences of external causes (S00-T98)	66.3 (*30*.*0%)*	45.6 (*28*.*0%)*	42.7 (*27*.*0%)*	45.6	55.5
External causes of morbidity and mortality (U50-Y98)	4.6 (*2*.*1%)*	3.9 (*2*.*4%)*	1.7 (*1*.*1%)*	17.1	173.3
Factors influencing health status and contact with health services (Z00-Z99)	32.4 (*14*.*6%)*	21.0 (*12*.*9%)*	16.3 (*10*.*3%)*	54.5	98.6

There was an increase in the average daily percentage presentation of non-indigenous patients consistent with the general increase in activity. In contrast attendances by those of indigenous status (Aboriginal and/or Torres Strait Islander) increased much less than the general increase and reached levels of significance (Χ^2^ = 12.63 p = .013). (Table 2)

While there was an increase in all Australasian Triage Scale categories except Category 1 (Resuscitation) it was most marked in the lower acuity Category 4 and Category 5. This did not have a significant impact on the distribution of attendances by triage categories. There was a trend toward decreased higher acuity presentations (Cat 1–3) and increased lower acuity presentations (Cat 4–5) (Χ^2^ = 52.11 p < .001). (Table 2)

While numbers increased the actual percentage admission and discharge rate remained consistent, as did rates for those who left without being seen or after treatment started. The only change of importance was a decrease in the proportion of patients being transferred to another hospital. (Table 2)

The most common diagnostic presentations were consistent across all years. Injury (S00-T98) was the most common in all three years while the next most common were Z00-Z99 (Factors influencing health care) and R00-R99 (Abnormal clinical and laboratory findings). Together with respiratory, infections, skin problems and genito-urinary presentations these made up approximately 70% of attendances in all years.

There were however, differences amongst ICD-10 codes in the level of variation from the average increase in attendances. Looking only at those diagnostic groupings which accounted for more than 2% of presentations, there were proportionally greater increases in attendances by those with skin problems (L00-L99) and factors influencing health care status (Z00-Z99) while there were proportionally less attendances by those with mental and behavioural disorders (F00-F99), diseases of the genito-urinary system (N00-N99) and abnormal clinical and laboratory findings (R00-R99). (Table 3)

## Discussion

This study describes the patterns of attendance to an Australian hospital following a tropical cyclone. There was an increase in presentations to the ED of TTH, which peaked in the first 24–48 hours following the cyclone and returned to normal over a five-day period. The 372 attendances on the 4-Feb-2011 were to our knowledge the highest number of presentations in a single day to any ED in Australia at this time, in 2012–13 the average number of presentations per hospital per day were for Australia were 105.7 (range 79.7 in the NT to 130.3 in Queensland[13]. The changes in presentations were mostly an amplification of normal attendance patterns with some altered areas of activity.

This amplification of normal attendance patterns was seen in the temporal patterns of attendance, the gender distribution of patients attending, the triage category proportions and admission and discharge rates. There were proportionally more presentations by the elderly, which increased with advancing age and by those with illness involving the skin or problems accessing health care. Of interest was the proportional decrease in attendance by those with mental health and behavioural disorders, those with genito-urinary problems and abnormal clinical and laboratory findings and those of indigenous status.

The general increase may be accounted for by exacerbations of pre-existing illness and disrupted access to alternative health services for both acute and chronic health care. This is consistent with experiences in other countries and health systems [14]. We did not specifically ask patients if they would have normally presented elsewhere but the peak in presentations correlates with the lack of availability of other health services in Townsville.

Virtually all General Practices were closed on the 2^nd^ and 3^rd^ February with a staggered re-introduction of services. This lack of alternative service providers may also be reflected in the increase in patients who presented with ICD-10 code Z00-Z99 (Factors influencing health status and contact with health services). This increase in presentations by those reliant on health services is most likely to represent those with existing, or chronic, disease who are also most likely to be the elderly.

The increasing presentations closely correlate with those of advancing age. Most presentations in this grouping reliant on access to health services, were related to closure of community dialysis facilities, loss of home oxygen supplies or inability of home nursing services to continue to visit. This is similar to the US experience following the closure of 94 dialysis facilities along the Gulf Coast.[15] Older people were also disproportionately affected following Hurricane Katrina[16]. In contrast however disruptions in the treatment of chronic disease were significantly more common among the non-elderly,[17] and the main increase in presentations of chronic disease related to endocrine, cardiovascular, and psychiatric disorders.[18] This is in contrast to our findings in which there was a decrease in mental health presentations and no change in the others. This may be due to a number of reasons including differences between populations in the studies, such as age, community disease profiles and levels of preparedness, and the period included in the study. We have only included data from the initial period post-Yasi to review the immediate impact on acute care. The impact of exacerbations of chronic disease or the effects on mental health may take longer to become evident dependent upon the disease process. The period of study would need to be extended to determine this, which is the subject of further work. It is interesting to note a US study found increased mortality for up to 2 months following four hurricanes in Florida with indirect mortality accounting for most deaths with major causes being heart (34%) and cancer-related deaths (19%).[19] We had no increase in presentations in either of these groups in our study.

In this study there were increased presentations with injury but not out of proportion to the general increase. While this is consistent with other developed countries, it was not seen to the same extent in which minor injuries have been observed to constitute over 80% of presentations following tropical cyclones.[20–22] There is however significant variation in the patterns between events, as well inconsistency in the classification and coding of injuries.[22] The most frequently reported mechanisms are falls and cuts,[23] (15) while there may also be an increase in the incidence of animal bites and stings.[20, 24, 25]

Infectious disease outbreaks are not a strong feature of tropical cyclones.[20, 25–27] If they do occur it is more likely to be in developing countries than in developed environments, and almost always manifest in the post-impact phase.[20, 28] The risk is associated with the size, health status and living conditions of the affected population with crowding, inadequate water and sanitation, as well as poor access to health services, increasing the risk of disease transmission.[26–28] Interestingly there was some variation in our findings around infectious diseases and while there was an increase in the ICD-10 grouping A00-B99 (Certain Infectious and Parasitic Diseases) for both years, only the 2010 / Yasi met the 50% increase criteria (predominantly for gastrointestinal infections) and further work is required exploring infection post-cyclones. It suggests emergency departments have an important role in monitoring presentations with gastrointestinal infections as part of a disease early warning system especially when people are evacuated to centres [29].

Cyclones also carry an increased risk of acute respiratory infection.[26, 27] While there was no increase in ARI in this study, this has occurred in the setting of multiple cases among a single population,[30] and possibly in association with increased environmental exposures.[31] This study had an increased number of presentations with skin infections. While theoretically sound, there is surprisingly little previous published data to support this. Following Hurricane Katrina there were reports of increased *Vibrio* species wound infections, mostly in patients with associated co-morbidities, who had been wading in flood-waters.[32]

There were no deaths directly attributed to Yasi in this study. However this study has not looked at the long-term health impacts of tropical cyclones or examined indirect mortality.

The findings of this study have implications for disaster preparedness of not just emergency departments but the health system as a whole. Knowledge of the likely patterns of injury and illness following a disaster helps inform not just surge capacity planning but also measures which can be taken in advance to support vulnerable community members and potentially prevent their need to present to hospital. We would argue that adding General Practice expertise to a hospital’s disaster response, where the disaster shuts all services in an areas would be worthwhile addition to the disasters services.

### Limitations

This was a single centre study of a single population and for one cyclone. Further studies are needed to see if these attendance patterns are maintained in other Australian populations and whether these differ with the severity of the cyclone involved and level of community preparedness. While this was a single centre study it should be noted that there is only one public hospital ED in Townsville with no other major hospital for over 300 km, which increases the validity of the data and the importance of the findings. This study also describes only the first week of activity following Yasi. Further studies are needed to address issues such as the impact of Yasi on mental health and psychological well-being as well as the potential for delayed presentations of either injury or illness, particularly infectious disease. Potential preparation time may have also impacted on the presentation as Yasi was the second cyclone to have threatened the region in a very short time period. There was also an annual increase in presentation to the TTH ED from 2009 to 2010, which we have tried to adjust for by have a criteria of a greater than 50% increase in cases.

## Conclusion

There was an increase in presentations to the ED of TTH, which peaked in the first 24–48 hours following the cyclone and returned to normal over a five-day period. The changes in presentations were mostly an amplification of normal attendance patterns with some altered areas of activity. Injury patterns are similar to overseas experience.

This research will help to guide future disaster planning arrangements, particularly in the areas of medical devices; medications and access to other services / stockpiles; injury prevention; staffing and support services.
